# Longitudinal Associations of Pubertal Timing and Tempo With Adolescent Mental Health and Risk Behavior Initiation in Urban South Africa

**DOI:** 10.1016/j.jadohealth.2020.09.043

**Published:** 2021-07

**Authors:** Alysse J. Kowalski, O. Yaw Addo, Michael R. Kramer, Reynaldo Martorell, Shane A. Norris, Rachel N. Waford, Linda M. Richter, Aryeh D. Stein

**Affiliations:** a Laney Graduate School, Emory University, Atlanta, Georgia; b SAMRC/WITS Developmental Pathways for Health Research Unit, Faculty of Health Sciences, University of the Witwatersrand, Johannesburg, South Africa; c Hubert Department of Global Health, Rollins School of Public Health, Emory University, Atlanta, Georgia; d Department of Epidemiology, Rollins School of Public Health, Emory University, Atlanta, Georgia; e DSI-NRF Centre of Excellence in Child Development, University of the Witwatersrand, Johannesburg, South Africa; f Department of Psychiatry and Behavioral Sciences, School of Medicine, Emory University, Atlanta, Georgia

**Keywords:** Pubertal timing, Pubertal tempo, Tanner sexual maturation scale, Menarche, Adolescent health, Low- and middle-income countries, Health risk behavior, Socioemotional adjustment, Eating attitudes, Birth to Twenty Plus

## Abstract

**Purpose:**

In high-income countries, early and rapid pubertal development is consistently associated with poor adjustment and increased risk behavior in adolescence. This study contributes to the meager knowledge of these associations in lower income countries.

**Methods:**

We used longitudinal data from 1,784 urban black South Africans in the Birth to Twenty Plus cohort. We used regression analyses to assess associations between age at menarche and latent classes of pubertal timing and tempo and adolescent internalizing and externalizing emotional and behavioral problems, eating attitudes, and patterns of health risk behavior initiation.

**Results:**

Relatively earlier and faster pubertal timing and tempo were associated with increased health risk behavior initiation (e.g., adjusted odds ratio [95% confidence interval] high- vs. low-risk pattern = 5.7 [1.7, 19.06] for male genital development; adjusted odds ratio = 3.45 [1.13, 10.49] for female breast development). Among males, earlier and faster pubertal timing and tempo were associated with increased externalizing problems in early adolescence and increased oppositional defiant problems in midadolescence, whereas later and slower pubertal timing and tempo were associated with decreases. Among females, earlier and faster pubertal timing and tempo were associated with increased internalizing and externalizing problems in midadolescence and increased dieting behaviors in early and late adolescence (β [95% confidence interval] = 2.51 [.87, 4.15] for pubic hair development), whereas later and slower pubertal timing and tempo were associated with decreases.

**Conclusions:**

In this urban South African cohort, relatively earlier and faster pubertal development was detrimental to mental health and risk behavior activity, whereas later and slower maturation was somewhat protective.

**Implications and Contribution**

Similar associations of pubertal timing and tempo with adolescent mental health and risk behavior initiation seen in high-income countries were observed in South Africa, a middle-income country. Early maturers in low- and middle-income countries may benefit from efforts to promote mental health and prevent and mitigate risk behaviors in adolescence.

The second decade of life is a stage of profound physical, cognitive, and socioemotional development [1]. Puberty, the development of secondary sex characteristics and reproductive capacity, unfolds alongside increases in height and changes in body composition, with variation in pubertal *timing*, or age of onset and *tempo*, or rate of progression having implications for adolescent health [2]. Relatively early timing has consistently been shown to be detrimental for a wide array of adolescent health outcomes, including internalizing problems, externalizing problems, disordered eating, substance use and abuse, and sexual behavior in cross-sectional and longitudinal studies [[3], [4], [5], [6], [7], [8]]. Late pubertal timing has been associated with psychopathology in selected studies, although this association was null in a 2017 meta-analysis [8]. Associations of pubertal tempo with adolescent health are less consistent, although faster tempo has been shown to be negatively associated with internalizing problems, including depressive symptoms, externalizing problems, and age at sexual debut in girls and boys [[9], [10], [11], [12]]. When considered together, early timing and faster tempo further exacerbated externalizing problems in boys, whereas early timing and slower tempo were associated with reduced substance use in girls [10,13].

These associations with adolescent health arise through the complex interplay of biological, cognitive, emotional, and social factors. Physical maturation alters social interactions such that adolescents may be treated in accordance with their physical appearance rather than their age with early and rapid maturers less cognitively and socioemotionally equipped to handle these changes [2]. Emotional regulation also develops later in adolescence such that young people may be more inclined toward impulsive and attention-seeking behaviors earlier on [14]. Beyond outward differences in physical appearance, there is some evidence suggesting early maturers secrete pubertal hormones at different concentrations, which may heighten sensitivity to environmental conditions and interactions [15,16].

Studies of pubertal timing and tempo have been conducted largely in high-income countries (HICs) and predominantly with non-Hispanic white youth. In HIC settings, pubertal timing differs by racial/ethnic subgroups within the population. In the U.S., black youth mature earliest, followed by their Latinx and non-Hispanic white peers [17,18]. Childhood environments characterized by high levels of psychosocial stressors, such as trauma (e.g., war), parental mental illness, parental absence, residence in a stepfamily, marital discord, harsh parental discipline, or absence of familial warmth, have been associated with accelerated pubertal timing [19]. Early life adversity has also been associated with poor psychosocial adjustment in adolescence [20]. Within a population, associations of pubertal timing and tempo with adolescent health are also modified by environmental factors such as neighborhood and school conditions [19,21,22]. Low- and middle-income countries (LMICs) have more poverty, inequality, adversity, and dual burdens of under- and over-nutrition than do HICs [23]. Moreover, because puberty is a biopsychosocial transition, the lens through which puberty is perceived by the adolescent and their community is influenced by cultural and contextual norms, which may themselves be shifting in response to urbanization and globalization [[24], [25], [26]]. Furthering our understanding of the implications of pubertal timing and tempo for health in different contexts is a worthwhile inquiry.

To this end, we examine associations of pubertal timing and tempo with several measures of adolescent health, including emotional and behavioral problems, eating attitudes, and health risk behavior initiation for boys and girls in urban South Africa. We further examine whether these associations differ with exposure to stressful events in childhood. Improving our understanding of these associations will inform public health policies and programs to support adolescent health in South Africa and may be informative to other LMICs.

## Methods

### Birth to Twenty Plus cohort

We analyzed data from the Birth to Twenty Plus (Bt20+) study, an observational cohort in Soweto-Johannesburg, South Africa. Inclusion criteria included a singleton birth between April and June 1990 and residence in the Soweto-Johannesburg municipal area for a minimum of 6 months after birth (N = 3,273) [27]. Participants were recruited from a combination of antenatal clinics, public delivery centers, and postnatal clinics within the predefined geographical area [27]. Most attrition took place within the first 2 years of life, and 72% of participants were traceable at age 16 years [28].

### Ethical approval

This study was approved by the University of the Witwatersrand Human Research Ethics Committee for Research on Human Subjects and the Emory University Institutional Review Board.

### Data collection

The initial visit took place at antenatal clinics and delivery centers. Subsequent study visits took place at intervals of 1–2 years and were conducted at the Developmental Pathways for Health Research Unit at Chris Hani Baragwanath academic hospital in Soweto. Participants were provided with snacks and reimbursed for transportation for each study visit. Questionnaires (emotional and behavioral problems, eating attitudes, and sexual maturation) and direct measurement (height and weight) were completed by trained research assistants. Self-administered questionnaires were used for sensitive topics (risk behaviors). Relevant measures and ages they were collected are summarized in Supplemental Table S1.

### Exposures

Pubertal development was assessed using the Tanner sexual maturation scale, a commonly used method for evaluating pubertal stage [29,30]. Tanner stages range from 1 (prepubertal) to 5 (postpubertal) with Stage 2 considered the onset of puberty. From ages 9–11 years, Tanner stage was assessed annually by a trained health care provider in a subset of participants. From ages 12–16 years, Tanner stage was self-assessed in the entire cohort after a local validation study found a high concordance between expert and self-assessment [31]. Age at menarche was asked at each adolescent study visit as part of the self-administered questionnaire, with no comparable measure for males.

**Figure 1:**
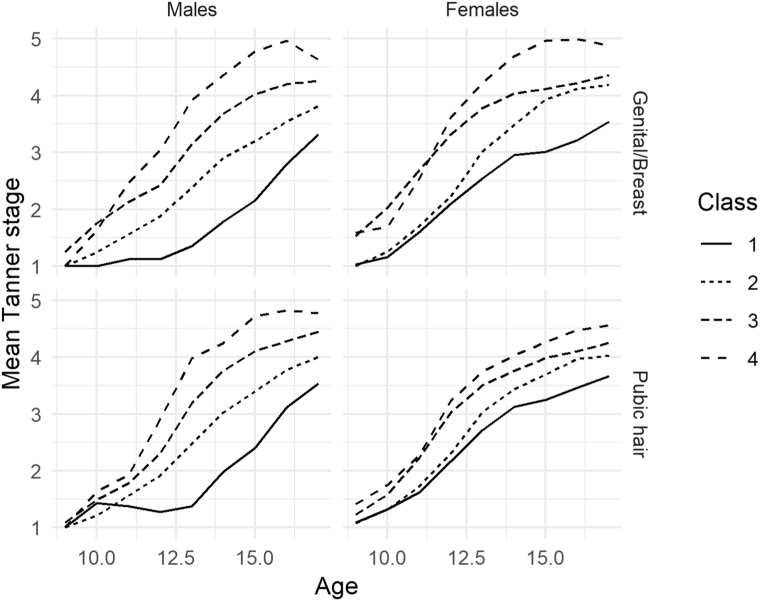
Mean Tanner stage development over time by latent classes of genital/breast and pubic hair development^a^. ^a^Latent classes derived by Lundeen EA, Norris SA, Martorell R, et al., redrawn for Black African cohort members [29].

Previously, sex-specific latent class growth analysis was used to group participants into three classes of pubic hair development and four classes of genital/breast development (Figure 1) [32]. The latent classes have similar shapes but are mostly nonoverlapping, differing with respect to age at pubertal onset and rate of progression to Stage 5. For each trajectory, Class 1 has the smallest intercept reflecting later pubertal onset and smallest slope reflecting slower progression. Age of onset is increasingly younger, and the rate of maturation is increasingly faster with each successive class such that individuals in the highest class (Class 3 for pubic hair development and Class 4 for genital/breast development) had the earliest pubertal timing and fastest tempo in the cohort. Latent classes were used to allow for potential nonlinear associations with the outcomes.

### Outcomes

For both sexes, three patterns of health risk behavior initiation (low, moderate, and high risk) were previously identified from timing of smoking, alcohol, cannabis, illicit drug, and sexual activity initiation through age 18 years [33]. Briefly, using annual reports of smoking, alcohol, marijuana, illicit drug use, and sexual activity in adolescence, we used hierarchical agglomerative cluster analysis to group participants into patterns of health risk behavior initiation. In general, individuals in the high-risk pattern initiated risk behaviors, including marijuana and illicit drug use, at higher rates than the group mean. Individuals in the moderate-risk pattern initiated smoking, alcohol use, and sexual activity at higher rates than the group mean but did not use marijuana. Individuals in the low-risk pattern initiated behaviors at lower rates than the group mean.

The 1991 Achenbach System of Empirically Based Assessment Youth Self-Report (YSR) was administered at ages 11 and 14 years to assess emotional and behavioral problems, which can impair mental health [34]. Questions were administered in English by trained interviewers who translated the questions into standardized phrases in local languages as needed. Participant responses were scored using six DSM-oriented scales from the 2001 version, three of which examined internalizing problems (affective problems, anxiety problems, and somatic problems) and three of which examined externalizing problems (attention deficit/hyperactivity problems, oppositional defiant problems, and conduct problems). We omitted the four items that were revised and one item that was inadvertently not administered. Items used in the DSM-oriented scales were rated as being “very consistent” with DSM-4 diagnostic criteria by international experts and have been shown to have good internal consistency and test–retest reliability in a diverse sample [35]. The YSR has been administered previously in South Africa, and the DSM-oriented scales have shown consistency when applied cross-culturally [36,37].

The Eating Attitudes Test (EAT) is widely used to screen for eating disorder risk and has three subscales (dieting, bulimia and food preoccupation, and oral control), which measure symptoms and concerns typical of eating disorders [38]. The 26-item EAT (EAT-26) was administered at ages 13 and 17 years. The EAT-26 had good internal reliability in early and late adolescence and has previously been applied in urban South Africa [39]. For both the YSR and EAT-26, clinical thresholds have not been validated in the South African context. We report mean scores for each scale, with higher scores reflecting increased problems or poorer eating attitudes.

### Covariates

Household asset ownership was used as a measure of socioeconomic position at two time points—age 0–2 years and age 7 years (supplemented with observations from age 5 years). We calculated tertiles from the number of assets owned. We used maternal reports of stress and violence events experienced in the past 6 months at the age 0–2, 5, and 7 years study visits as proxies of childhood stress exposure. Events captured included experiences of intimate partner, political, and community violence, familial stress (e.g., family member death, illness, or conflict with the law), child stress (e.g., separation from child and inability to find childcare), and economic hardship. We defined a composite childhood stress variable as the number of visits in which the mother reported experiencing above the sample median number of events. Height-for-age z-scores and body mass index for age z-scores at ages 5 and 8 years were used as measures of child growth [40].

### Analytical sample

We restricted our analytical sample to 1,784 black African cohort members for whom we have a pubertal development trajectory and allowed the sample size to vary for each outcome to maximize the available data (Supplemental Figure S1). Black Africans are the largest population group in Soweto-Johannesburg and comprise 78% of the Bt20+ cohort.

### Two-way imputation

For the YSR, EAT-26, asset ownership, and childhood stress measures, we used two-way imputation, a deterministic imputation approach that uses the person mean and the sample item mean to assign a response value to a missing item for individuals who completed at least 50% of the relevant items [41]. The EAT-26 completion rate was 54% at age 17 years, and imputation recovered 24 observations. The household asset ownership completion rate was 64% from Years 0 to 2, and imputation recovered 308 observations. Two-way imputation was applied to other measures for consistency although completion rates were ≥90%.

### Statistical analysis

We used multinomial logistic regression to examine associations of the pubertal timing and tempo measures (genital/breast development classes, pubic hair development classes, and age of menarche) with risk behavior initiation pattern using the low-risk pattern as the referent group in the outcome. The exponentiated coefficients can be interpreted as the relative odds of being classified in a particular risk behavior pattern compared with the low-risk pattern. We used linear regression to examine associations of the pubertal timing and tempo measures with the three internalizing problem scales, the three externalizing problem scales, and eating attitudes. In all regression models, Class 2 pubertal development (moderate timing and tempo) was the referent group. We controlled for household socioeconomic position in early life and childhood and childhood stress. Height-for-age z-scores and body mass index for age z-scores at ages 5 and 8 years were not consistently associated with the outcomes or exposure in bivariate analyses and were not adjusted for. All analyses were sex specific.

We assessed the heterogeneity of the pubertal timing and tempo estimates across categories of childhood stress by examining interaction *p* values. Considering all models, we identified associations that would survive a 10% false discovery rate. We interpreted the main results holistically and focused on those suggesting a pattern of associations that holds across multiple indicators of pubertal timing and tempo or multiple time points. All analyses were conducted using R version 3.5.3 with two-sided *p* values <.05 considered statistically significant [42].

## Results

The mothers of participants included in the analysis had more years of schooling and higher asset ownership, reported more stressful events, and were more likely to be single at the child's birth than those excluded (Supplemental Table S2.). In males, 6% were in the later and slower genital development class (Class 1), and 7% were in the earliest and fastest class (Class 4), which differed from females, where 23% were in the later and slower breast development class and 14% were in the earliest and fastest class (Table 1). Distribution across the pubic hair development classes was more similar between the sexes. For genital development, pubertal timing ranged from age 11.6 years in Class 4–14.4 years in Class 1, whereas the timing of pubic hair development ranged from age 11.9 years in Class 3–13.3 years in Class 1 among males. For breast development, pubertal timing ranged from age 11.0 years in Class 4–12.3 years in Class 1, whereas the timing of pubic hair development ranged from age 10.8 years in Class 3–13.0 years in Class 1 among males (Figure 1).

**Table 1:** Characteristics of birth to Twenty Plus analytical sample

	Measure	Males (N)	Males, n (%)	Females (N)	Females, n (%)
Household assets in early life in early life, tertile	1 (lowest)	700	299 (43%)	764	308 (40%)
	2		155 (22%)		203 (27%)
	3 (highest)		246 (35%)		254 (33%)
Early life assets imputed	Yes	857	107 (12%)	917	133 (14%)
Household assets in early life at age 7 y, tertile	1 (lowest)	708	307 (43%)	786	337 (43%)
	2		192 (27%)		200 (25%)
	3 (highest)		209 (30%)		250 (32%)
Age 7 y asset source	Age 7 y	708	689 (97%)	786	747 (95%)
Child stress, number of study waves in which above-median events were reported	None	785	341 (43%)	856	340 (40%)
	One		283 (36%)		337 (39%)
	Two or three		161 (21%)		180 (21%)
Number of stress inventories completed	1	785	148 (19%)	856	149 (17%)
	2		351 (45%)		420 (49%)
	3		286 (36%)		288 (34%)
BMIZ 5 y^a^		585	.33 (.96)	651	.21 (.91)
HAZ 5 y^a^		585	−.68 (.92)	651	−.64 (.91)
BMIZ 8 y^a^		486	−.04 (.9)	493	−.03 (.96)
HAZ 8 y^a^		488	−.62 (1)	493	−.69 (.94)
Genital (males)/breast (females) development class^b^	1 (Latest and slowest)	861	55 (6%)	923	212 (23%)
	2 3		316 (37%) 429 (50%)		229 (25%) 351 (38%)
	4 (Earliest and fastest)		61 (7%)		131 (14%)
Pubic hair development class^b^	1 (Latest and slowest)	861	253 (29%)	923	310 (34%)
	2		504 (59%)		499 (54%)
	3 (Earliest and fastest)		104 (12%)		114 (12%)
Age at Genital (males)/breast (females) development onset^c^	y	861	12.7 (1.5)	923	12.3 (1.4)
Age at pubic hair development onset^c^	y	861	12.6 (1.3)	923	12.5 (1.4)
Age at menarche	y	--	--	906	12.7 (1.22)

BMIZ = body mass index for age z-scores; HAZ = height-for-age z-scores.

a Based on World Health Organization Child Growth Standards. Presented as mean (SD).

b Latent classes derived from repeated measures of the Tanner sexual maturation scale [19]. Individuals in Class 1 started puberty later and progressed through puberty slower than their peers in other classes and individuals in the highest class had the earliest pubertal timing and fastest tempo.

c Age at which Tanner stage 2 was reached.

Among individuals who reported initiating a risk behavior, the mean ages of smoking, alcohol, and sexual activity initiation were lower among males than females, with mean age of sexual activity initiation over a year earlier among males (age 15.3 years) compared with females (age 16.6 years; Table 2). One third of males and 17% of females followed the high-risk behavior initiation pattern, initiating both an increased number of risk behaviors and doing so at a younger age, on average. Anxiety and somatic problem scores decreased from 11 to 14 years, whereas attention deficit, oppositional defiant, and conduct problem scores increased (Table 2). Except for conduct problems, the mean scores on the internalizing and externalizing scales were slightly higher for females than males at both ages. Female eating attitude scores increased slightly from 9.9 (7.7) at age 13 years–11.1 (8.7) at age 17 years, whereas male scores were unchanged.

**Table 2:** Descriptive characteristics of adolescent emotional and behavior adjustment, eating attitudes, and risk behavior initiation

	Measure	Males, N	Males, mean (SD)	Females, N	Females, mean (SD)
Risk behavior initiation pattern^a^	Low risk	498	169 (34%)	569	130 (23%)
	Moderate risk		157 (32%)		341 (60%)
	High risk		172 (35%)		98 (17%)
Age of first smoke	y	711	13.3 (2.5)	643	14.5 (2.3)
Age of first alcohol use	y	534	14.0 (2.3)	516	14.4 (2.4)
Age of first marijuana use	y	245	14.8 (.9)	126	14.6 (1.1)
Age of first illicit drug use	y	272	16.2 (1.7)	133	16.3 (1.9)
Age of first sexual activity	y	571	15.3 (1.7)	621	16.6 (1.4)
Age 11 y emotional and behavioral problem scores					
Affective problems	Range 0–26	565	3.3 (2.4)	624	3.5 (2.8)
Anxiety problems	Range 0–12	565	3.7 (1.8)	616	3.9 (1.8)
Somatic problems	Range 0–14	572	2.9 (2.2)	629	3.9 (2.3)
Attention deficit problems	Range 0–14	573	2.6 (2.5)	630	3.5 (2.7)
Oppositional defiant problems	Range 0–10	572	1.3 (1.5)	630	1.6 (1.6)
Conduct problems	Range 0–30	570	2.5 (2.6)	630	2.2 (2.2)
Age 14 y emotional and behavioral problem scores					
Affective problems	Range 0–26	565	3.2 (2.7)	624	4.2 (3.5)
Anxiety problems	Range 0–12	565	3.2 (1.8)	616	3.3 (1.9)
Somatic problems	Range 0–14	572	.6 (1.6)	629	.9 (2.1)
Attention deficit problems	Range 0–14	573	3.3 (2.9)	630	4.1 (2.8)
Oppositional defiant problems	Range 0–10	572	1.8 (1.9)	630	2.4 (2)
Conduct problems	Range 0–30	570	3.1 (3.1)	630	2.6 (2.6)
Age 13 y eating attitude scores					
Total eating attitudes	Range 0–78	463	10 (7.1)	487	9.9 (7.7)
Dieting	Range 0–39	463	5.1 (4.1)	487	5 (4.4)
Bulimia and food preoccupation	Range 0–18	463	1.7 (2.5)	487	1.4 (2.3)
Oral control	Range 0–21	463	3.2 (2.9)	487	3.5 (3.5)
Age 17 y eating attitude scores					
Total eating attitudes	Range 0–78	463	10 (6.7)	487	11.1 (8.7)
Dieting	Range 0–39	463	5.2 (4.4)	487	5.9 (5.5)
Bulimia and food preoccupation	Range 0–18	463	1.5 (2.2)	487	1.6 (2.6)
Oral control	Range 0–21	463	3.3 (2.9)	487	3.6 (3.6)

a Risk behavior patterns derived from a hierarchical cluster analysis of adolescent smoking, alcohol, cannabis, illicit drug, and sexual activity initiation [21]. Presented as n (%).

### Males

#### Risk behavior initiation

Among males, earlier and more rapid genital development was associated with increasing odds of following the moderate- or high-risk behavior pattern compared with low-risk behavior pattern (Class 3 vs. 2: moderate adjusted odds ratio [AOR] = 1.86 [1.03, 3.34]; high AOR = 2.42 [1.37, 4.28]; class 4 vs. 2: moderate AOR = 5.61 [1.65, 19.07]; high AOR = 5.7 [1.7, 19.06]; Table 3, Supplemental Figure S2). Later and slower pubic hair development was associated with decreased odds of following the moderate- and high-risk behavior pattern compared with low-risk pattern (pubic hair development Class 1 vs. 2: moderate AOR = .34 [.18, .64]; high AOR = .62 [.35, 1.09]).

**Table 3:** Adjusted associations of male genital and pubic hair development classes with adolescent emotional and behavioral adjustment, eating attitudes, and patterns of risk behavior initiation^a^

	Gen Class 1	Gen Class 2	Gen Class 3	Gen Class 4	PH Class 1	PH Class 2	PH Class 3
	Later onset and slower tempo			Earlier onset and faster tempo	Later onset and slower tempo		Earlier onset and faster tempo
Pattern of risk behavior initiation	OR (95% CI)	Ref	OR (95% CI)	OR (95% CI)	OR (95% CI)	Ref	OR (95% CI)
Low risk	Ref	Ref	Ref	Ref	Ref	Ref	Ref
Moderate risk	.85 (.23, 3.15)	Ref	1.86 (1.03, 3.34)	5.61 (1.65, 19.07)^b^	.34 (.18, .64)^b^	Ref	1.66 (.68, 4.09)
High risk	1.01 (.3, 3.46)	Ref	2.42 (1.37, 4.28)^b^	5.7 (1.7, 19.06)^b^	.62 (.35, 1.09)	Ref	2.17 (.92, 5.14)
Age 11 y sociobehavioral adjustment	β (95% CI)	Ref	β (95% CI)	β (95% CI)	β (95% CI)	Ref	β (95% CI)
Aff	.16 (−.91, 1.22)	Ref	−.07 (−.58, .44)	.37 (−.54, 1.28)	−.09 (−.62, .44)	Ref	.09 (−.64, .81)
Anx	1.25 (.42, 2.08)^b^	Ref	.28 (−.12, .68)	−.26 (−.97, .45)	−.35 (−.77, .06)	Ref	−.4 (−.97, .17)
Som	−.86 (−1.84, .11)	Ref	.18 (−.29, .64)	.11 (−.73, .94)	−.56 (−1.04, −.08)	Ref	.46 (−.2, 1.12)
Attn Def	.15 (−1, 1.3)	Ref	.25 (−.31, .8)	.44 (−.55, 1.42)	−.53 (−1.09, .04)	Ref	.48 (−.3, 1.26)
Opp Def	−.33 (−1.01, .36)	Ref	.11 (−.22, .44)	.44 (−.14, 1.03)	−.38 (−.72, −.04)	Ref	.22 (−.25, .68)
Conduct	−.18 (−1.33, .98)	Ref	−.02 (−.57, .52)	.85 (−.12, 1.82)	−.24 (−.8, .33)	Ref	.86 (.09, 1.63)
Age 14 y sociobehavioral adjustment	β (95% CI)	Ref	β (95% CI)	β (95% CI)	β (95% CI)	Ref	β (95% CI)
Aff	.88 (−.34, 2.11)	Ref	.8 (.17, 1.42)	−.22 (−1.32, .88)	−.48 (−1.11, .15)	Ref	−1.26 (−2.1, −.41)^b^
Anx	−.13 (−.98, .73)	Ref	−.08 (−.5, .35)	−.13 (−.89, .63)	−.19 (−.61, .24)	Ref	−.13 (−.71, .45)
Som	.47 (−.29, 1.24)	Ref	.25 (−.14, .63)	.09 (−.59, .77)	−.05 (−.44, .34)	Ref	.01 (−.52, .54)
Attn Def	1.27 (−.01, 2.55)	Ref	.66 (.02, 1.3)	.52 (−.61, 1.66)	−.45 (−1.1, .21)	Ref	−.75 (−1.64, .13)
Opp Def	−.3 (−1.14, .54)	Ref	.64 (.23, 1.06)^b^	1.16 (.43, 1.89)^b^	−.62 (−1.04, −.19)^b^	Ref	.42 (−.15, .99)
Conduct	.15 (−1.26, 1.56)	Ref	.95 (.24, 1.66)^b^	−.04 (−1.29, 1.2)	−1.07 (−1.78, −.35)^b^	Ref	−.67 (−1.64, .3)
Age 13 y eating attitudes	β (95% CI)	Ref	β (95% CI)	β (95% CI)	β (95% CI)	Ref	β (95% CI)
Total	−.72 (−3.75, 2.32)	Ref	−.61 (−2.06, .84)	−.78 (−3.28, 1.73)	−.42 (−1.92, 1.07)	Ref	−.33 (−2.32, 1.65)
Dieting	.04 (−1.68, 1.75)	Ref	−.34 (−1.16, .48)	−.24 (−1.66, 1.18)	−.15 (−.99, .7)	Ref	.4 (−.72, 1.52)
Bulimia	.09 (−.97, 1.16)	Ref	.24 (−.27, .74)	.15 (−.73, 1.03)	−.11 (−.63, .42)	Ref	−.13 (−.82, .57)
Oral control	−.84 (−2.04, .35)	Ref	−.51 (−1.08, .06)	−.69 (−1.68, .3)	−.17 (−.76, .42)	Ref	−.6 (−1.39, .18)
Age 17 y eating attitudes	β (95% CI)	Ref	β (95% CI)	β (95% CI)	β (95% CI)	Ref	β (95% CI)
Total	−.11 (−3.09, 2.87)	Ref	−1.57 (−3.17, .04)	1.18 (−1.6, 3.95)	2.1 (.45, 3.74)	Ref	2.14 (−.02, 4.29)
Dieting	−.01 (−1.97, 1.94)	Ref	−1 (−2.06, .05)	1.02 (−.8, 2.84)	1.21 (.12, 2.29)	Ref	1.07 (−.35, 2.49)
Bulimia	−.22 (−1.2, .76)	Ref	−.23 (−.76, .3)	.42 (−.5, 1.33)	.26 (−.28, .8)	Ref	.62 (−.09, 1.33)
Oral control	.13 (−1.12, 1.37)	Ref	−.34 (−1.01, .33)	−.26 (−1.42, .9)	.63 (−.06, 1.31)	Ref	.44 (−.46, 1.34)

Aff = affective problems; Anx = anxiety problems; Attn Def = attention deficit problems; CI = confidence interval; Conduct = conduct problems; Gen Class = genital development class; Opp Def = oppositional defiant problems; OR = odds ratio; PH Class = pubic hair development class; Ref = reference; Som = somatic problems.

a Pattern of risk behavior initiation estimates are adjusted odds ratios from multinomial logistic regression. All other estimates are beta coefficients from linear regression. Models adjusted for household asset ownership at age 0–2 y, household asset ownership at age 7 y, childhood stress exposure, and companion variables for each measure.

b Association significant given 10% false discovery rate.

#### Internalizing problems, externalizing problems, and eating attitudes

At age 11 years, earlier and faster genital and pubic hair development were each associated with small increases in attention deficit problems, oppositional defiant problems, and conduct problems, whereas later and slower development were associated with small, although not statistically significant, decreases in these externalizing problems (Table 3, Supplemental Figure S3). At age 14 years, earlier and faster genital and pubic hair development were each associated with increased oppositional defiant problems, and later and slower genital and pubic hair development were each associated with decreased oppositional defiant problems (e.g., genital development Class 4 vs. 2: β = 1.16 [.43, 1.89]; Class 1 vs. 2: β = −.3 [−1.14, .54]). There was no pattern of association across the other externalizing scales. There was no evidence that genital or pubic hair timing and tempo were associated with affective problems, anxiety problems, or somatic problems at ages 11 or 14 years or eating attitudes at age 13 or 17 years (Table 3, Supplemental Figures S3 and S4).

### Females

#### Risk behavior initiation

Among females, earlier and more rapid breast development was associated with increasing odds of following the moderate-risk (Class 3 vs. 2 AOR = 1.8 [.97, 3.34]; Class 4 vs. 2 AOR = 1.99 [.89, 4.44]) or high-risk (Class 3 vs. 2 AOR = 3.44 [1.38, 8.58]; Class 4 vs. 2 AOR = 3.45 [1.13, 10.49]) patterns compared with the low-risk pattern (Table 4, Supplemental Figure S5). Similarly, relatively later and slower pubic hair development was associated with decreasing odds of following the moderate-risk (Class 1 vs. 2 AOR = .58 [.35, .95]) or high-risk (Class 1 vs. 2 AOR = .54 [.27, 1.1]) patterns compared with the low-risk pattern as was later age at menarche (moderate-risk AOR = .82 [.66, 1.0]; high AOR = .53 [.39, .71]).

**Table 4:** Adjusted associations of female breast and pubic hair development classes and age at menarche with adolescent emotional and behavioral adjustment, eating attitudes, and patterns of risk behavior initiation^a^

	Br Class 1	Br Class 2	Br Class 3	Br Class 4	PH Class 1	PH Class 2	PH Class 3	Age at menarche
	Later onset and slower tempo			Earlier onset and faster tempo	Later onset and slower tempo		Earlier onset and faster tempo	
Pattern of risk behavior initiation	OR (95% CI)	Ref	OR (95% CI)	OR (95% CI)	OR (95% CI)	Ref	OR (95% CI)	OR (95% CI)
Low risk	Ref	Ref	Ref	Ref	Ref	Ref	Ref	Ref
Moderate risk	.88 (.46, 1.66)	Ref	1.8 (.97, 3.34)	1.99 (.89, 4.44)	.58 (.35, .95)	Ref	1.49 (.62, 3.63)	.82 (.66, 1)
High risk	1.25 (.45, 3.45)	Ref	3.44 (1.38, 8.58)^b^	3.45 (1.13, 10.49)	.54 (.27, 1.1)	Ref	2.12 (.74, 6.04)	.53 (.39, .71)^b^
Age 11 y sociobehavioral adjustment	β (95% CI)	Ref	β (95% CI)	β (95% CI)	β (95% CI)	Ref	β (95% CI)	β (95% CI)
Aff	−.36 (−1.12, .4)	Ref	−.65 (−1.31, .01)	−.6 (−1.43, .22)	.41 (−.15, .97)	Ref	.13 (−.64, .89)	−.11 (−.32, .1)
Anx	.2 (−.29, .7)	Ref	−.05 (−.48, .38)	.12 (−.42, .66)	.06 (−.3, .43)	Ref	.01 (−.49, .51)	−.02 (−.16, .11)
Som	−.25 (−.91, .4)	Ref	.05 (−.52, .62)	.11 (−.6, .83)	−.14 (−.62, .34)	Ref	−.39 (−1.05, .27)	−.12 (−.3, .06)
Attn Def	−.57 (−1.33, .19)	Ref	.02 (−.64, .68)	0 (−.83, .83)	.24 (−.31, .8)	Ref	.46 (−.31, 1.22)	−.19 (−.4, .02)
Opp Def	.06 (−.35, .47)	Ref	.18 (−.18, .53)	−.04 (−.49, .41)	−.12 (−.42, .18)	Ref	.39 (−.02, .8)	−.1 (−.22, .01)
Conduct	−.26 (−.88, .35)	Ref	−.09 (−.63, .44)	−.14 (−.81, .53)	.18 (−.27, .63)	Ref	.16 (−.45, .77)	−.16 (−.33, .01)
Age 14 y sociobehavioral adjustment	β (95% CI)	Ref	β (95% CI)	β (95% CI)	β (95% CI)	Ref	β (95% CI)	β (95% CI)
Aff	.11 (−.88, 1.1)	Ref	.32 (−.55, 1.19)	.72 (−.39, 1.84)	.21 (−.53, .95)	Ref	1.03 (.02, 2.05)	−.29 (−.58, 0)
Anx	−.27 (−.82, .28)	Ref	−.01 (−.5, .47)	.04 (−.58, .66)	−.44 (−.85, −.03)	Ref	.31 (−.26, .87)	−.12 (−.28, .04)
Som	−.11 (−.69, .48)	Ref	−.03 (−.54, .49)	.39 (−.27, 1.05)	−.23 (−.67, .21)	Ref	−.29 (−.89, .32)	−.13 (−.3, .04)
Attn Def	−.67 (−1.45, .1)	Ref	.35 (−.33, 1.03)	.37 (−.51, 1.24)	.09 (−.5, .67)	Ref	1.11 (.31, 1.92)^b^	−.47 (−.7, −.24)^b^
Opp Def	−.47 (−1.02, .07)	Ref	−.05 (−.53, .42)	−.23 (−.84, .38)	−.22 (−.63, .19)	Ref	.21 (−.35, .77)	−.19 (−.35, −.03)
Conduct	−.21 (−.94, .52)	Ref	.48 (−.16, 1.12)	.69 (−.13, 1.51)	−.4 (−.94, .15)	Ref	.32 (−.44, 1.07)	−.16 (−.38, .05)
Age 13 y eating attitudes	β (95% CI)	Ref	β (95% CI)	β (95% CI)	β (95% CI)	Ref	β (95% CI)	β (95% CI)
Total	1.85 (−.04, 3.74)	Ref	.36 (−1.33, 2.05)	1.93 (−.18, 4.04)	1.37 (−.03, 2.78)	Ref	1.56 (−.4, 3.53)	.18 (−.38, .74)
Dieting	.58 (−.48, 1.63)	Ref	.71 (−.23, 1.66)	2.12 (.94, 3.3)^b^	.14 (−.65, .93)	Ref	1.37 (.27, 2.48)	−.11 (−.43, .2)
Bulimia	.39 (−.16, .95)	Ref	−.31 (−.81, .18)	−.67 (−1.29, −.04)	.63 (.22, 1.05)^b^	Ref	.05 (−.54, .63)	.1 (−.06, .27)
Oral control	.88 (.04, 1.72)	Ref	−.03 (−.78, .72)	.48 (−.46, 1.41)	.6 (−.02, 1.23)	Ref	.15 (−.73, 1.02)	.19 (−.06, .44)
Age 17 y eating attitudes	β (95% CI)	Ref	β (95% CI)	β (95% CI)	β (95% CI)	Ref	β (95% CI)	β (95% CI)
Total	−.91 (−3.45, 1.63)	Ref	.2 (−2.18, 2.58)	.28 (−2.6, 3.16)	1.7 (−.23, 3.64)	Ref	4.58 (1.92, 7.24)^b^	−.32 (−1.11, .47)
Dieting	−.63 (−2.19, .93)	Ref	−.07 (−1.53, 1.39)	.73 (−1.04, 2.5)	1.04 (−.16, 2.24)	Ref	2.51 (.87, 4.15)^b^	−.14 (−.63, .34)
Bulimia	−.33 (−1.08, .43)	Ref	−.24 (−.95, .47)	−.82 (−1.68, .04)	.27 (−.32, .86)	Ref	.79 (−.01, 1.6)	.05 (−.19, .28)
Oral control	.04 (−1.01, 1.1)	Ref	.51 (−.47, 1.5)	.37 (−.82, 1.56)	.39 (−.42, 1.2)	Ref	1.27 (.17, 2.38)	−.23 (−.55, .1)

Aff = affective problems; Anx = anxiety problems; Attn Def = attention deficit problems; Br class = breast development class; CI = confidence interval; Conduct = conduct problems; Opp Def = oppositional defiant problems; OR = odds ratio; PH class = pubic hair development class; Ref = reference; Som = somatic problems.

a Pattern of risk behavior initiation estimates are adjusted odds ratios from multinomial logistic regression. All other estimates are beta coefficients from linear regression. Models adjusted for household asset ownership at age 0–2 y, household asset ownership at age 7 y, childhood stress exposure, and companion variables for each measure.

b Association significant given 10% false discovery rate.

#### Internalizing problems, externalizing problems, and eating attitudes

At age 11 years, there was no evidence that breast or pubic hair development onset was associated with internalizing problems (Table 4, Supplemental Figure S6). At age 14 years, relatively earlier and faster pubic hair development was associated with a 1.03 (.02, 2.05) point increase in affective problem score, whereas later age at menarche was associated with a −.29 (−.58, 0) point decrease in affective problem score; although not significant, breast development followed a similar pattern. In general, earlier and faster maturation was associated with small increases in anxiety and somatic problems, and later and slower maturation was associated with small decreases.

Although there was no evidence that breast and pubic hair development were associated with externalizing problems at age 11 years, with few exceptions earlier, and faster pubertal development was associated with increased attention deficit problems, oppositional defiant problems, and conduct problems at age 14 years, and problem scores decreased if development was relatively later and slower (Table 4, Supplemental Figure S7). For example, relatively earlier and faster pubic hair development (Class 3 vs. 2) was associated with a 1.11 (.31, 1.92) point increase in attention deficit score, and older age at menarche was associated with a −.47 (−.7, −.24) point decrease.

Age 13 and 17 years, dieting behaviors were increased in the earliest and fastest breast and pubic hair development classes (e.g., age 13 years pubic hair development Class 3 vs. 2 β = 1.37 [.27, 2.48]) and declined with decreasing development class and increasing age at menarche (Table 4, Supplemental Figure S8).

Unadjusted model results can be found in Supplemental Tables S3 and S4. We found three statistically significant interactions between childhood stress exposure and associations of pubertal development and adolescent adjustment among males and two significant interactions among females, within the expected number of type I errors (Supplemental Table S5). Of the significant findings, 46% remain so at a 10% false discovery rate (52% of among males and 39% among females).

## Discussion

We used multiple high-quality measures of pubertal timing and tempo and rich longitudinal data on adolescent emotional and behavioral health and risk behavior initiation to paint a comprehensive picture of the consequences of offset maturation during adolescence in urban South Africa. We found that generally, earlier and faster maturation was detrimental to emotional and behavioral health and risk behavior activity, whereas later and slower maturation was slightly protective.

Earlier and more rapid pubertal development was associated with above-average rates and earlier initiation of smoking, alcohol, and sexual activity, and the earliest and fastest maturers were more likely to experiment with cannabis and illicit drug use. These findings are consistent with systematic reviews and meta-analyses that have shown positive associations between early pubertal timing and both substance use and earlier age of intercourse [3,[6], [7], [8]]. Although imprecise, our estimates were large and similar in magnitude to results from a Finnish study in which earlier age at menarche was associated with multiple indicators of smoking, alcohol, and illegal drug use in midadolescence [43]. Adolescent behavior takes cues from the social environment and may differ by race/ethnicity and by gender. In one study of African American youth, early maturing girls were more likely to use substances, whereas in a separate study, early maturing nonwhite boys did not engage in increased substance use [44,45]. In Bt20+, later and slower maturation was slightly protective of risk behavior initiation, which is inconsistent with a meta-analysis that found no effect [8].

In meta-analyses by Dimler and Natsuaki and Ullsperger and Nikolas, early pubertal timing was associated with small increases in externalizing behaviors for both sexes, as were the increases we observed [4,8]. Ullsperger and Nikolas did not find differences by behavior assessment age, whereas in Bt20+, we observed both sex and age differences. We observed small protective associations for late maturation with externalizing behaviors although this was not observed in the Ullsperger meta-analysis [8]. In a study of early adolescence, early maturing African American youth reported increased externalizing behaviors, which were further amplified by harsh and inconsistent parenting practices and neighborhood conditions [21].

Pubertal timing and tempo were associated with a small increase in internalizing problems in mid, although not early, adolescence among females, and there was no evidence of an association in males. Ullsperger and Nikolas also found that early pubertal timing had a small positive effect on internalizing symptoms, although their finding did not differ by sex [8]. In a study of African American youth, early maturation was consistently associated with depressive symptoms in girls, whereas associations for boys abated after early adolescence [46]. Earlier and faster maturing females reported increased unhealthy eating attitudes, consistent with studies that have shown early pubertal timing, was associated with increased disordered eating symptoms and rates of eating disorders [5].

The maturational disparity hypothesis offers a developmental explanation for the consistency of our findings with those from HICs. The hypothesis states that early maturers of both sexes are at increased risk of psychological distress because of the mismatch between their physical development and slower progressing cognitive and emotional development, which may contribute to risk behavior through increased sensation seeking desire or socialization with older peers [47]. The consequences of earlier maturation may be more severe for females who risk becoming pregnant and who are twice as likely to experience adolescent-onset internalizing disorders during puberty [48].

Counter to our expectations, we did not find evidence of statistical interactions between pubertal timing and tempo and childhood stress exposure. Although stressful early life environments are associated with earlier pubertal timing, the associated stressors reflect direct or highly proximal interaction with the developing child (i.e., maltreatment and parent–child relationship quality) [19,22,49]. It is possible that associations with these types of stressors were obscured by other dimensions of stress that were endorsed at high levels (i.e., economic hardship) or that maternal experiences may not be an adequate proxy for her child's experience. Different dimensions of stress should be partitioned in future work.

This is a longitudinal study of salient adolescent health outcomes conducted in an LMIC, which is underrepresented in the literature. An additional strength is the use of high-quality Tanner sexual maturation scale measures and latent classes of pubertal timing and tempo that identify subgroups of earlier/faster and later/slower maturers for both sexes. We were likely underpowered for this analysis and subsequently have focused on patterns of associations that can be observed across multiple measures of pubertal timing and tempo and interpreted the results holistically. This contributed to small cell counts and imprecise estimates for some of the latent classes (Class 4 genital development in particular). Our results are not generalizable to population subgroups beyond black South Africans, although black South Africans are the dominant population subgroup.

Our results suggest that early maturing urban South African adolescents may benefit from interventions at the individual, family, or peer levels during the pubertal transition [50]. For example, individual-level interventions that strengthen coping strategies and problem-solving before adolescence may allow the early maturer to better navigate puberty [50]. In South Africa's 2017 National Adolescent and Youth Health Policy, drug and substance use, mental health, and sexual and reproductive health were identified as priority areas in which evidence-based interventions are being enacted by the National Department of Health [51]. Interventions in these priority areas may have additional benefits to early maturers.

Associations of pubertal timing and tempo with adolescent emotional and behavioral health and risk behavior initiation in urban South Africa were broadly consistent in direction and magnitude with those observed in high-income settings. Future research should confirm these findings and examine pathways by which these associations arise and factors that may modify them.
