# Supplementary material for: Longitudinal Associations of Pubertal Timing and Tempo With Adolescent Mental Health and Risk Behavior Initiation in Urban South Africa
Source: J Adolesc Health. 2021 Jul;69(1):64–73. doi: 10.1016/j.jadohealth.2020.09.043 (PMC8253008; doi:10.1016/j.jadohealth.2020.09.043)

Longitudinal associations of pubertal timing and tempo with adolescent mental health and risk behavior initiation in urban South Africa

Supplemental Tables and Figures

Supplemental Table S1. Selected measures and data collection waves

	Study wave										
	0-2 y	5 y	7 y	9 y	11 y	13 y	14 y	15 y	16 y	17 y	18 y
Sexual maturity scale				X	X	X	X	X	X	X	X
Age of menarche				X	X	X	X	X	X	X	X
Emotional & behavioral problems					X		X				
Eating attitudes						X				X	
Smoking					X	X	X	X	X	X	X
Alcohol use					X	X					X
Cannabis use					X	X	X	X			
Drug use					X	X	X	X		X	X
Sexual activity					X	X	X	X	X	X	X
HH asset ownership	X	X	X								
Child stress exposure	X	X	X								

Supplemental Table S2. Descriptive characteristics of included and excluded participants

	Included (n = 1784)	Excluded (n = 784)	p-value
Sex - Females	922 (52%)	397 (51%)	0.66
Maternal age at birth	25.77 (6.24)	26.03 (5.94)	0.33
Maternal years of schooling	9.63 (2.71)	8.46 (3.59)	< 0.01
Marital status - Single	1186 (67%)	445 (57%)	< 0.01
- Partnered	591 (33%)	337 (43%)	
Asset tertile in early life - 1	610 (34%)	301 (38%)	< 0.01
- 2	360 (20%)	89 (11%)	
- 3	503 (28%)	85 (11%)	
- Missing	311 (17%)	309 (39%)	
Asset tertile at age 7 y - 1	647 (36%)	95 (12%)	< 0.01
- 2	395 (22%)	36 (5%)	
- 3	461 (26%)	50 (6%)	
- Missing	281 (16%)	603 (77%)	
Child stress in early life - Below median	490 (27%)	218 (28%)	< 0.01
- Above median	397 (22%)	133 (17%)	
- Missing	897 (50%)	433 (55%)	
Child stress at age 5 y - Below median	738 (41%)	91 (12%)	< 0.01
- Above median	436 (24%)	33 (4%)	
- Missing	610 (34%)	660 (84%)	
Child stress at age 7 y - Below median	907 (51%)	101 (13%)	< 0.01
- Above median	538 (30%)	39 (5%)	
- Missing	339 (19%)	644 (82%)	

Supplemental Table S3. Unadjusted associations of male genital and pubic hair development classes with adolescent emotional and behavioral adjustment, eating attitudes, and patterns of risk behavior initiation[a]

	Gen class 1 (Later and slower)	Gen class 2	Gen class 3	Gen class 4 (Earlier and faster)	PH class 1 (Later and slower)	PH class 2	PH class 3 (Earlier and faster)
Pattern of risk behavior initiation	OR (95% CI)	Ref	OR (95% CI)	OR (95% CI)	OR (95% CI)	Ref	OR (95% CI)
Low-risk	Ref	Ref	Ref	Ref	Ref	Ref	Ref
Moderate risk	1.17 (0.45, 3.01)	Ref	1.5 (0.94, 2.4)	3.56 (1.3, 9.77)	0.57 (0.35, 0.93)	Ref	2.25 (1.02, 4.99)
High-risk	1.17 (0.44, 3.11)	Ref	2.16 (1.35, 3.45)[b]	4.01 (1.46, 11.05)[b]	0.73 (0.45, 1.16)	Ref	2.52 (1.15, 5.51)
Age 11 y sociobehavioral adjustment	β (95% CI)	Ref	β (95% CI)	β (95% CI)	β (95% CI)	Ref	β (95% CI)
Aff	0.24 (-0.63, 1.11)	Ref	-0.08 (-0.51, 0.35)	0.27 (-0.56, 1.1)	-0.01 (-0.46, 0.44)	Ref	-0.06 (-0.71, 0.59)
Anx	0.6 (-0.05, 1.25)	Ref	0.27 (-0.06, 0.59)	-0.31 (-0.92, 0.3)	-0.25 (-0.59, 0.08)	Ref	-0.27 (-0.75, 0.21)
Som	-0.68 (-1.49, 0.12)	Ref	0.26 (-0.14, 0.65)	0.15 (-0.61, 0.9)	-0.4 (-0.81, 0.01)	Ref	0.38 (-0.21, 0.98)
Attn Def	0.04 (-0.88, 0.96)	Ref	0.38 (-0.07, 0.84)	0.41 (-0.46, 1.28)	-0.44 (-0.91, 0.03)	Ref	0.73 (0.06, 1.41)
Opp Def	-0.21 (-0.76, 0.34)	Ref	0.22 (-0.06, 0.49)	0.45 (-0.08, 0.97)	-0.31 (-0.6, -0.03)	Ref	0.37 (-0.04, 0.78)
Conduct	-0.33 (-1.3, 0.64)	Ref	-0.08 (-0.56, 0.39)	0.48 (-0.42, 1.38)	0.05 (-0.44, 0.54)	Ref	0.72 (0.02, 1.42)
Age 14 y sociobehavioral adjustment	β (95% CI)	Ref	β (95% CI)	β (95% CI)	β (95% CI)	Ref	β (95% CI)
Aff	1.15 (0.19, 2.12)	Ref	0.75 (0.26, 1.24)[b]	-0.12 (-1.05, 0.81)	-0.41 (-0.92, 0.09)	Ref	-0.97 (-1.67, -0.26)[b]
Anx	0.08 (-0.59, 0.76)	Ref	-0.13 (-0.46, 0.2)	-0.29 (-0.93, 0.35)	-0.08 (-0.42, 0.27)	Ref	-0.13 (-0.61, 0.36)
Som	0.35 (-0.22, 0.91)	Ref	0.07 (-0.21, 0.36)	0.06 (-0.48, 0.6)	-0.12 (-0.41, 0.17)	Ref	0.02 (-0.39, 0.43)
Attn Def	1.05 (0.01, 2.09)	Ref	0.52 (-0.01, 1.04)	0.51 (-0.49, 1.5)	-0.38 (-0.92, 0.16)	Ref	-0.52 (-1.28, 0.23)
Opp Def	-0.41 (-1.08, 0.25)	Ref	0.29 (-0.04, 0.62)	1.11 (0.48, 1.74)[b]	-0.47 (-0.81, -0.13)[b]	Ref	0.56 (0.09, 1.04)
Conduct	0.03 (-1.09, 1.15)	Ref	0.33 (-0.23, 0.9)	0.38 (-0.69, 1.45)	-0.4 (-0.98, 0.18)	Ref	-0.29 (-1.1, 0.52)
Age 13 y eating attitudes	β (95% CI)	Ref	β (95% CI)	β (95% CI)	β (95% CI)	Ref	β (95% CI)
Total	-0.29 (-2.72, 2.13)	Ref	-0.61 (-1.77, 0.54)	-0.71 (-2.83, 1.41)	-0.18 (-1.39, 1.02)	Ref	0.03 (-1.63, 1.7)
Dieting	-0.27 (-1.67, 1.14)	Ref	-0.21 (-0.88, 0.46)	-0.27 (-1.5, 0.96)	-0.15 (-0.85, 0.55)	Ref	0.46 (-0.5, 1.42)
Bulimia	0.43 (-0.43, 1.3)	Ref	0.24 (-0.17, 0.66)	0.27 (-0.49, 1.03)	-0.2 (-0.63, 0.23)	Ref	-0.01 (-0.6, 0.59)
Oral control	-0.46 (-1.45, 0.54)	Ref	-0.64 (-1.12, -0.17)[b]	-0.71 (-1.58, 0.16)	0.17 (-0.32, 0.66)	Ref	-0.42 (-1.1, 0.26)
Age 17 y eating attitudes	β (95% CI)	Ref	β (95% CI)	β (95% CI)	β (95% CI)	Ref	β (95% CI)
Total	0.85 (-1.59, 3.3)	Ref	-1.43 (-2.75, -0.1)	0.43 (-2.05, 2.91)	2.34 (0.98, 3.71)[b]	Ref	1.82 (-0.12, 3.77)
Dieting	0.42 (-1.18, 2.01)	Ref	-0.85 (-1.71, 0.01)	0.83 (-0.79, 2.45)	1.27 (0.38, 2.16)[b]	Ref	1.14 (-0.13, 2.41)
Bulimia	-0.28 (-1.09, 0.53)	Ref	-0.36 (-0.8, 0.08)	0.03 (-0.79, 0.85)	0.38 (-0.08, 0.83)	Ref	0.42 (-0.22, 1.07)
Oral control	0.72 (-0.33, 1.77)	Ref	-0.22 (-0.78, 0.35)	-0.43 (-1.49, 0.63)	0.7 (0.12, 1.29)	Ref	0.26 (-0.58, 1.1)

[a] Pattern of risk behavior initiation estimates are adjusted odds ratios from multinomial logistic regression. All other estimates are beta coefficients from linear regression. [b] Association significant given 10% false discovery rate. Abbreviations: Gen class, genital development class; PH class, pubic hair development class; Aff, affective problems; Anx, anxiety problems; Som, somatic problems; Attn Def, attention deficit problems; Opp Def, oppositional defiant problems; Conduct, conduct problems.

Supplemental Table S4. Unadjusted associations of female breast and pubic hair development classes and age of menarche with adolescent emotional and behavioral adjustment, eating attitudes, and patterns of risk behavior initiation[a]

	Br class 1 (Later and slower)	Br class 2 →	Br class 3 →	Br class 4 (Earlier and faster)	PH class 1 (Later and slower)	PH class 2 →	PH class 3 (Earlier and faster)	Age at menarche
Pattern of risk behavior initiation	OR (95% CI)	Ref	OR (95% CI)	OR (95% CI)	OR (95% CI)	Ref	OR (95% CI)	OR (95% CI)
Low-risk	Ref	Ref	Ref	Ref	Ref	Ref	Ref	Ref
Moderate risk	0.66 (0.38, 1.15)	Ref	1.4 (0.82, 2.39)	1.48 (0.72, 3.04)	0.53 (0.34, 0.82)[b]	Ref	1.31 (0.62, 2.76)	0.85 (0.72, 1.01)
High-risk	0.93 (0.42, 2.09)	Ref	2.21 (1.06, 4.61)	2.8 (1.12, 6.97)	0.6 (0.34, 1.08)	Ref	1.9 (0.8, 4.54)	0.62 (0.5, 0.79)[b]
Age 11 y sociobehavioral adjustment	β (95% CI)	Ref	β (95% CI)	β (95% CI)	β (95% CI)	Ref	β (95% CI)	β (95% CI)
Aff	-0.22 (-0.86, 0.42)	Ref	-0.63 (-1.19, -0.07)	-0.31 (-1.03, 0.4)	0.25 (-0.23, 0.73)	Ref	0.06 (-0.6, 0.72)	-0.11 (-0.28, 0.06)
Anx	0.11 (-0.3, 0.52)	Ref	-0.01 (-0.36, 0.35)	0.35 (-0.11, 0.81)	0.04 (-0.27, 0.34)	Ref	0.2 (-0.23, 0.62)	-0.04 (-0.15, 0.07)
Som	-0.2 (-0.74, 0.33)	Ref	0.04 (-0.42, 0.5)	0.24 (-0.35, 0.84)	-0.21 (-0.61, 0.19)	Ref	-0.17 (-0.72, 0.38)	-0.15 (-0.3, -0.01)
Attn Def	-0.49 (-1.12, 0.14)	Ref	0.15 (-0.4, 0.7)	0.31 (-0.4, 1.02)	0.1 (-0.38, 0.57)	Ref	0.61 (-0.05, 1.27)	-0.18 (-0.35, -0.01)
Opp Def	-0.05 (-0.41, 0.31)	Ref	0.05 (-0.27, 0.36)	0.08 (-0.32, 0.49)	-0.14 (-0.41, 0.13)	Ref	0.34 (-0.03, 0.71)	-0.16 (-0.25, -0.06)[b]
Conduct	0.1 (-0.41, 0.62)	Ref	-0.14 (-0.59, 0.31)	0.06 (-0.52, 0.64)	0.38 (-0.01, 0.77)	Ref	0.29 (-0.24, 0.82)	-0.05 (-0.19, 0.09)
Age 14 y sociobehavioral adjustment	β (95% CI)	Ref	β (95% CI)	β (95% CI)	β (95% CI)	Ref	β (95% CI)	β (95% CI)
Aff	0.03 (-0.78, 0.84)	Ref	0.22 (-0.49, 0.93)	0.98 (0.07, 1.9)	-0.12 (-0.73, 0.49)	Ref	1.04 (0.19, 1.89)	-0.39 (-0.62, -0.16)[b]
Anx	-0.11 (-0.56, 0.34)	Ref	-0.05 (-0.45, 0.35)	0.22 (-0.28, 0.73)	-0.38 (-0.72, -0.05)	Ref	0.22 (-0.25, 0.69)	-0.17 (-0.3, -0.05)[b]
Som	-0.16 (-0.64, 0.31)	Ref	-0.09 (-0.51, 0.33)	0.28 (-0.25, 0.82)	-0.18 (-0.54, 0.18)	Ref	-0.24 (-0.74, 0.26)	-0.08 (-0.21, 0.05)
Attn Def	-0.34 (-0.98, 0.3)	Ref	0.48 (-0.09, 1.04)	0.66 (-0.06, 1.39)	-0.15 (-0.63, 0.33)	Ref	1.29 (0.62, 1.96)[b]	-0.44 (-0.62, -0.27)[b]
Opp Def	-0.43 (-0.88, 0.03)	Ref	0.03 (-0.37, 0.43)	0.12 (-0.4, 0.64)	-0.42 (-0.77, -0.08)	Ref	0.38 (-0.1, 0.86)	-0.33 (-0.46, -0.2)[b]
Conduct	0 (-0.6, 0.59)	Ref	0.48 (-0.05, 1)	0.78 (0.1, 1.45)	-0.41 (-0.86, 0.04)	Ref	0.35 (-0.28, 0.98)	-0.18 (-0.35, -0.02)
Age 13 y eating attitudes	β (95% CI)	Ref	β (95% CI)	β (95% CI)	β (95% CI)	Ref	β (95% CI)	β (95% CI)
Total	0.96 (-0.64, 2.56)	Ref	0.21 (-1.21, 1.64)	1.29 (-0.51, 3.09)	0.9 (-0.31, 2.11)	Ref	0.67 (-1.02, 2.36)	-0.07 (-0.52, 0.38)
Dieting	0.23 (-0.67, 1.13)	Ref	0.84 (0.03, 1.64)	1.86 (0.85, 2.87)[b]	0.05 (-0.64, 0.73)	Ref	0.97 (0.01, 1.93)	-0.25 (-0.51, 0.01)
Bulimia	0.26 (-0.21, 0.74)	Ref	-0.18 (-0.6, 0.25)	-0.43 (-0.96, 0.11)	0.39 (0.03, 0.75)	Ref	-0.07 (-0.57, 0.43)	0.02 (-0.12, 0.15)
Oral control	0.47 (-0.26, 1.19)	Ref	-0.45 (-1.09, 0.2)	-0.15 (-0.96, 0.67)	0.46 (-0.09, 1.01)	Ref	-0.23 (-0.99, 0.54)	0.16 (-0.04, 0.37)
Age 17 y eating attitudes	β (95% CI)	Ref	β (95% CI)	β (95% CI)	β (95% CI)	Ref	β (95% CI)	β (95% CI)
Total	-0.35 (-2.61, 1.9)	Ref	-0.01 (-2.08, 2.06)	0.88 (-1.64, 3.4)	0.86 (-0.87, 2.59)	Ref	2.31 (-0.03, 4.65)	-0.33 (-1, 0.33)
Dieting	-0.04 (-1.44, 1.37)	Ref	-0.01 (-1.31, 1.28)	1.08 (-0.49, 2.65)	0.66 (-0.43, 1.74)	Ref	1.19 (-0.27, 2.65)	-0.13 (-0.54, 0.29)
Bulimia	-0.28 (-0.94, 0.38)	Ref	-0.24 (-0.85, 0.37)	-0.64 (-1.38, 0.1)	0.01 (-0.50, 0.53)	Ref	0.29 (-0.4, 0.98)	0.01 (-0.19, 0.21)
Oral control	-0.03 (-0.95, 0.89)	Ref	0.25 (-0.6, 1.09)	0.43 (-0.6, 1.46)	0.19 (-0.51, 0.90)	Ref	0.83 (-0.13, 1.79)	-0.22 (-0.49, 0.06)

[a] Pattern of risk behavior initiation estimates are adjusted odds ratios from multinomial logistic regression. All other estimates are beta coefficients from linear regression. [b] Association significant given 10% false discovery rate. Abbreviations: Br class, breast development class; PH class, pubic hair development class; Aff, affective problems; Anx, anxiety problems; Som, somatic problems; Attn Def, attention deficit problems; Opp Def, oppositional defiant problems; Conduct, conduct problems.

Supplemental Table S5. Associations of genital/breast development and pubic hair development classes with adolescent emotional and behavioral adjustment modified by childhood stress[a]

	Gen/Br class 1<br>β (95% CI)	Gen/Br class 2<br>β (95% CI)	Gen/Br class 3<br>β (95% CI)	Gen/Br class 4<br>β (95% CI)	PH class 1<br>β (95% CI)	PH class 2<br>β (95% CI)	PH class 3<br>β (95% CI)
Males							
Som 11 y 0X	--	--	--	--	-0.57 (-1.19, 0.05)	Ref	0.54 (-0.32, 1.4)
Som 11 y 1X	--	--	--	--	-1.17 (-1.94, -0.41)[b]	Ref	-1.33 (-2.52, -0.13)
Som 11 y 2-3X	--	--	--	--	0.36 (-0.46, 1.18)	Ref	1.75 (0.64, 2.87)[b]
Attn Def 14 y 0X	0.81 (-0.71, 2.33)	Ref	0.8 (-0.01, 1.6)	2.25 (0.6, 3.9)[b]	--	--	--
Attn Def 14 y 1X	0.77 (-0.85, 2.4)	Ref	-0.32 (-1.23, 0.59)	-0.37 (-1.95, 1.21)	--	--	--
Attn Def 14 y 2-3X	2.74 (-0.14, 5.62)	Ref	1.9 (0.63, 3.17)[b]	-0.24 (-2.38, 1.9)	--	--	--
Conduct 14 y 0X	--	--	--	--	-0.96 (-1.87, -0.05)	Ref	0.21 (-1.05, 1.47)
Conduct 14 y 1X	--	--	--	--	0.56 (-0.51, 1.64)	Ref	-0.93 (-2.45, 0.58)
Conduct 14 y 2-3X	--	--	--	--	-1.9 (-3.27, -0.53)[b]	Ref	-0.75 (-2.49, 0.98)
Females							
Attn Def 11 y 0X	-1.17 (-2.29, -0.04)	Ref	0.15 (-0.8, 1.11)	0.78 (-0.45, 2.02)	-0.05 (-0.84, 0.74)	Ref	1.63 (0.48, 2.77)[b]
Attn Def 11 y 1X	-0.99 (-1.95, -0.02)	Ref	-0.37 (-1.22, 0.49)	-0.91 (-1.94, 0.11)	0 (-0.75, 0.75)	Ref	-0.9 (-1.99, 0.18)
Attn Def 11 y 2-3X	1.37 (-0.03, 2.77)	Ref	1.23 (-0.01, 2.47)	1.68 (-0.15, 3.51)	0.83 (-0.25, 1.91)	Ref	1.03 (-0.3, 2.37)
Som 14 y 0X	0.15 (-0.62, 0.92)	Ref	0.29 (-0.37, 0.96)	1.34 (0.51, 2.18)[b]	--	--	--
Som 14 y 1X	-0.72 (-1.49, 0.06)	Ref	-0.51 (-1.2, 0.18)	-0.75 (-1.6, 0.09)	--	--	--
Som 14 y 2-3X	0.6 (-0.5, 1.7)	Ref	0.1 (-0.86, 1.06)	0.27 (-1.19, 1.73)	--	--	--

[a] Estimates are β (95% CI) from linear regressions. [b] Association significant given 10% false discovery rate. Abbreviations: Gen/Br class, genital/breast development class; PH class, pubic hair development class; Som, somatic problems; Attn Def, attention deficit problems; Conduct, conduct problems.

Longitudinal associations of pubertal timing and tempo with adolescent mental health and risk behavior initiation in urban South Africa

Supplemental Figure S1. Birth to Twenty Plus analytical sample

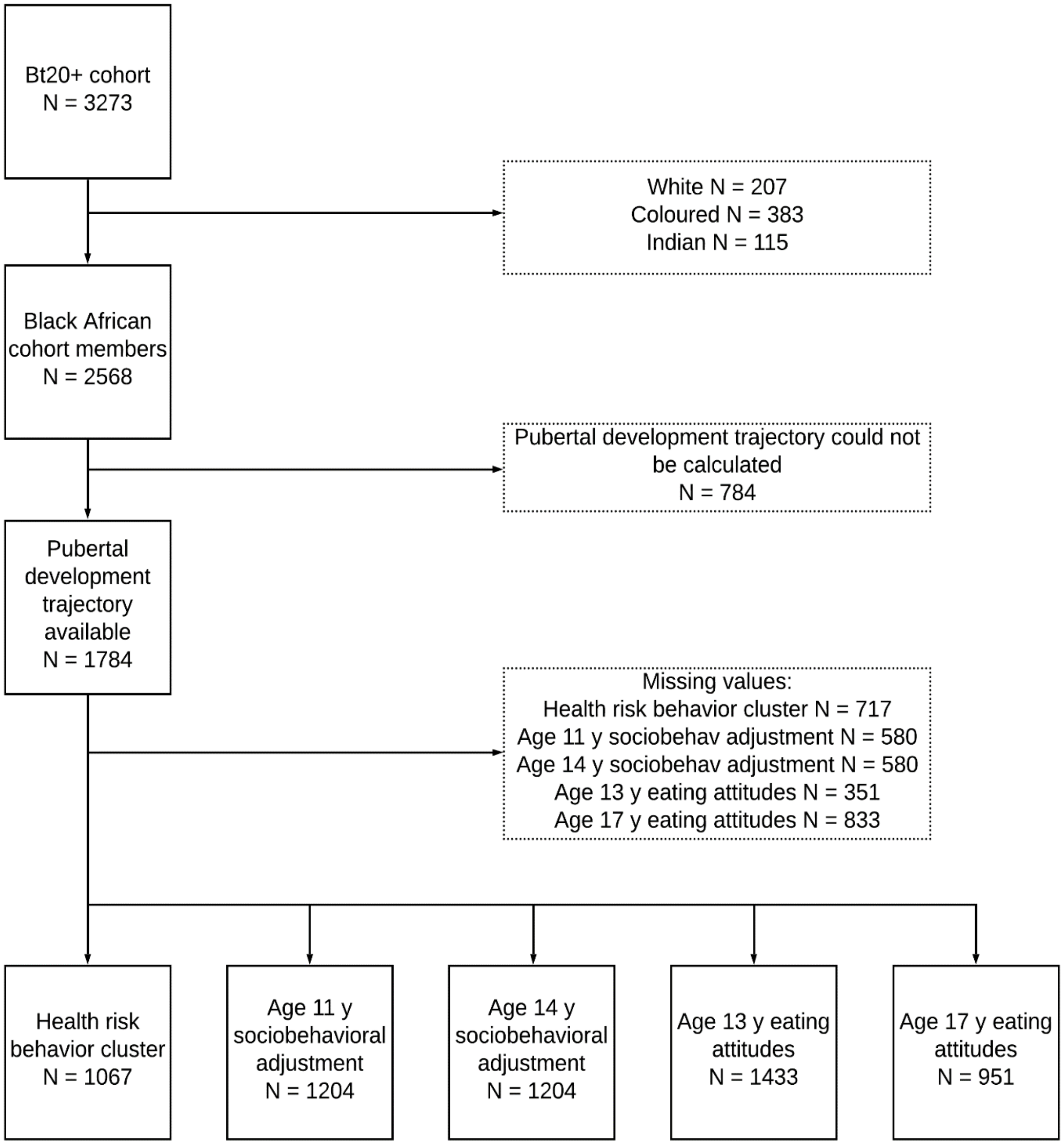

Longitudinal associations of pubertal timing and tempo with adolescent mental health and risk behavior initiation in urban South Africa

Supplemental Figure S2. Adjusted associations of male genital and pubic hair development classes with patterns of health risk behavior initiation

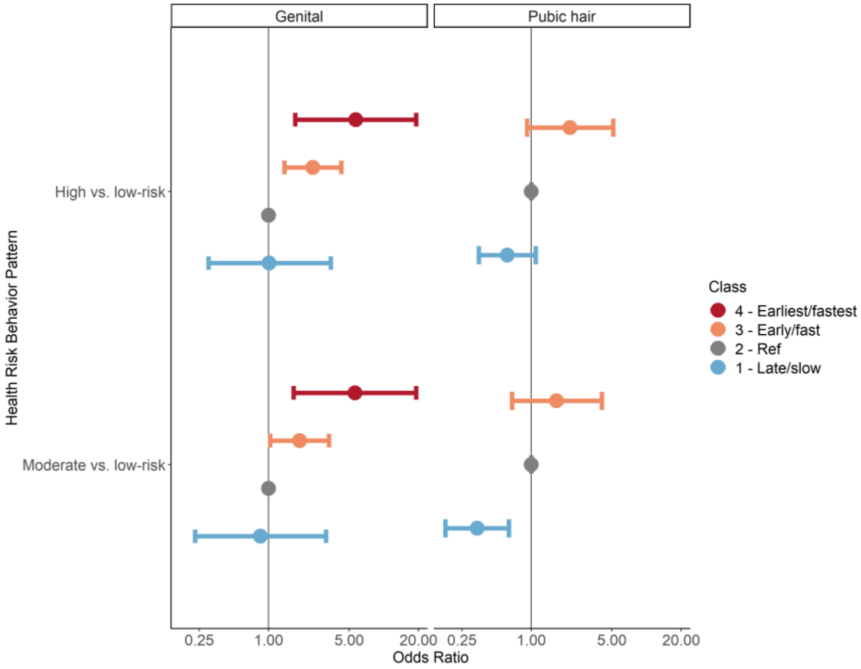

Longitudinal associations of pubertal timing and tempo with adolescent mental health and risk behavior initiation in urban South Africa

Supplemental Figure S3. Adjusted associations of male genital and pubic hair development classes with emotional and behavioral problems at ages 11 and 14 y

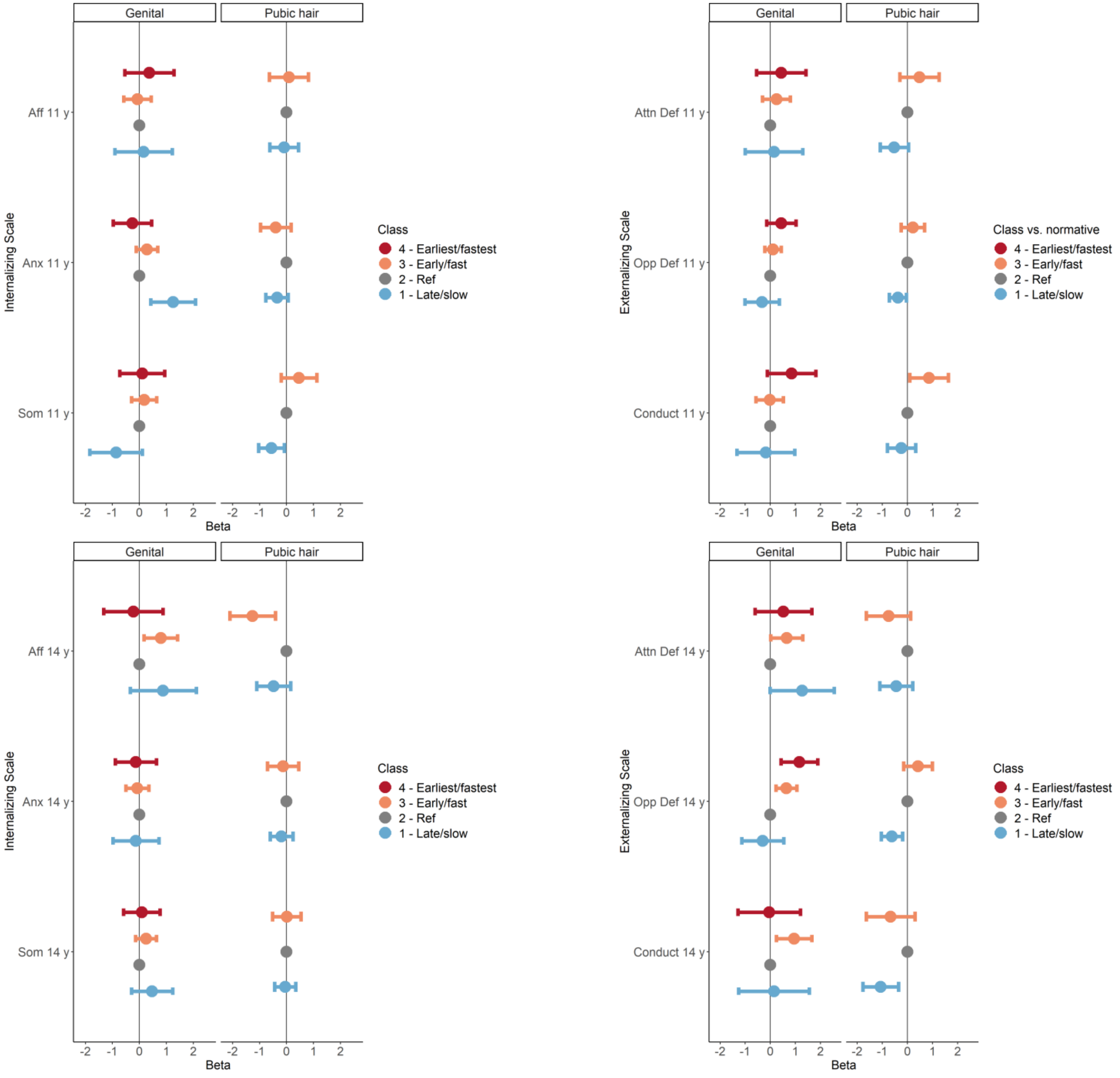

Longitudinal associations of pubertal timing and tempo with adolescent mental health and risk behavior initiation in urban South Africa

Supplemental Figure S4. Adjusted associations of male genital and pubic hair development classes with eating attitudes at ages 13 and 17 y

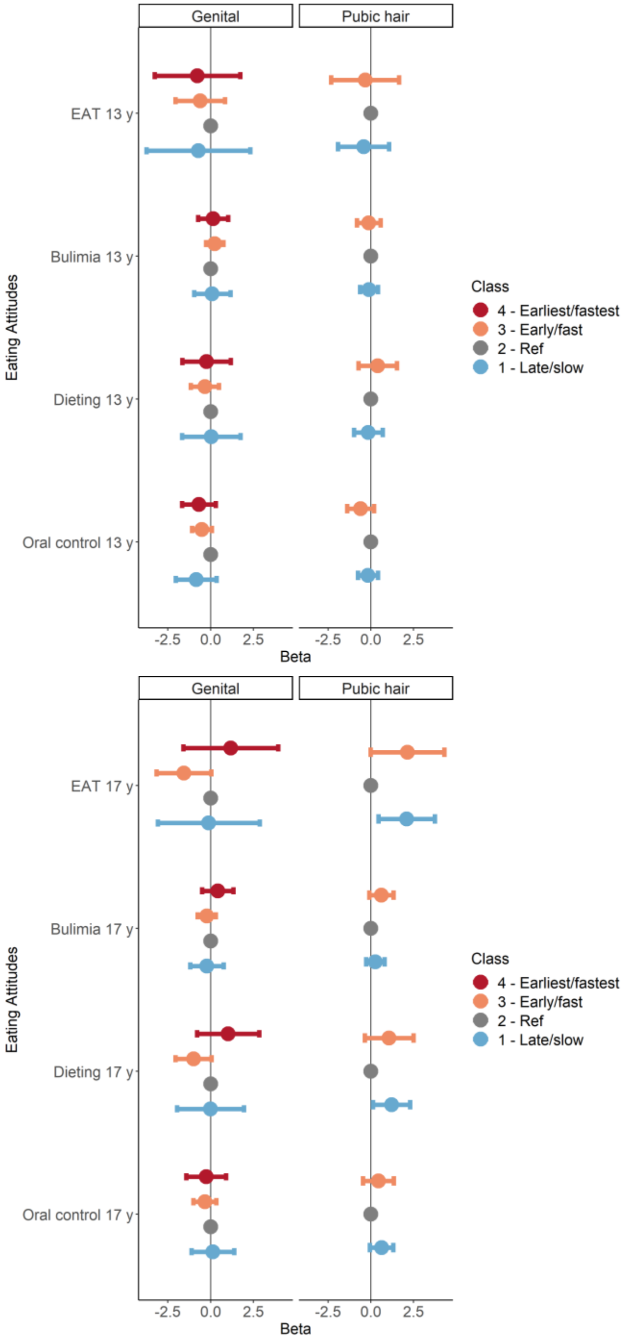

Longitudinal associations of pubertal timing and tempo with adolescent mental health and risk behavior initiation in urban South Africa

Supplemental Figure S5. Adjusted associations of female breast and pubic hair development classes and age at menarche with health risk behavior initiation patterns

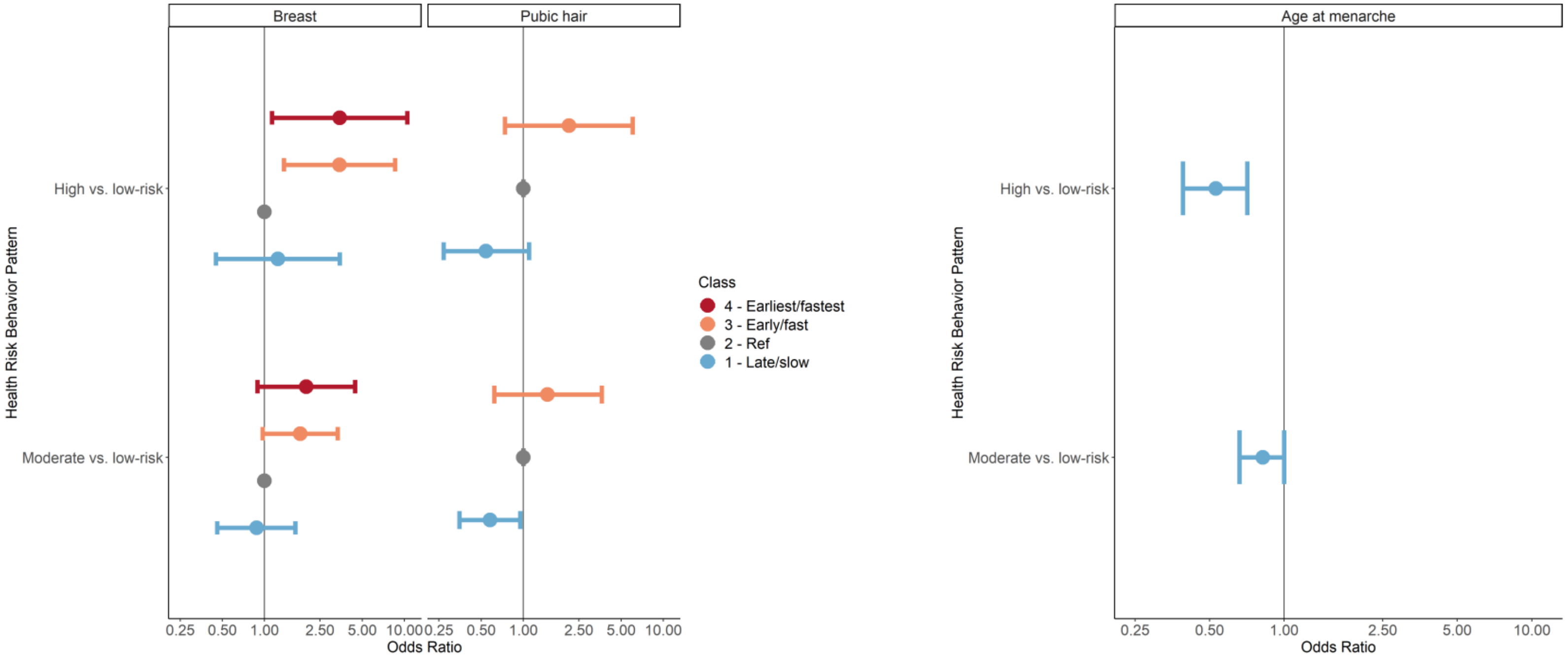

Longitudinal associations of pubertal timing and tempo with adolescent mental health and risk behavior initiation in urban South Africa

Supplemental Figure S6. Adjusted associations of female breast and pubic hair development classes and age at menarche with affective problems, anxiety problems, and somatic problems at ages 11 and 14 y

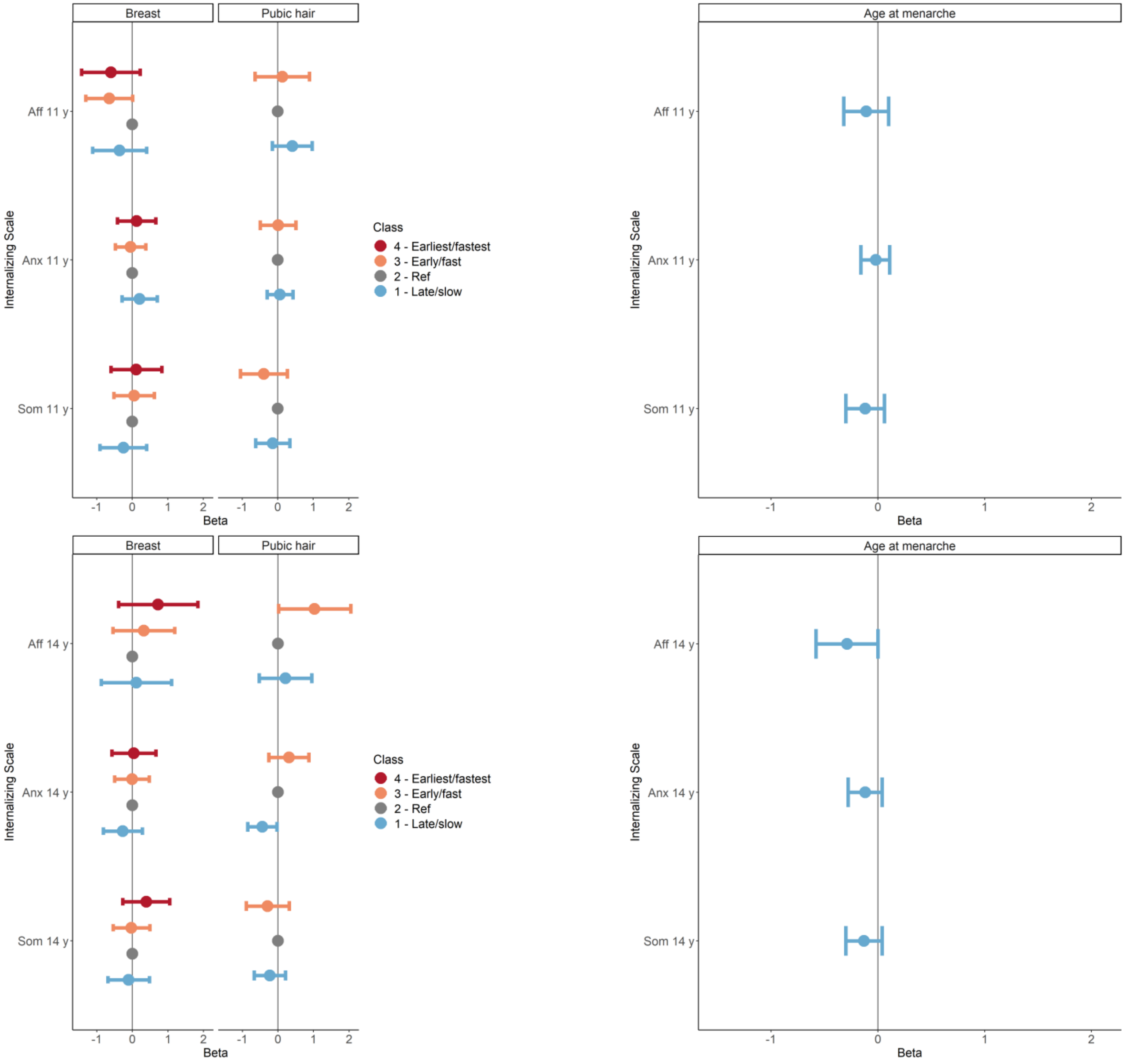

Supplemental Figure S7. Adjusted associations of female breast and pubic hair development classes and age at menarche with attention deficit problems, oppositional defiant problems, and conduct problems at ages 11 and 14 y

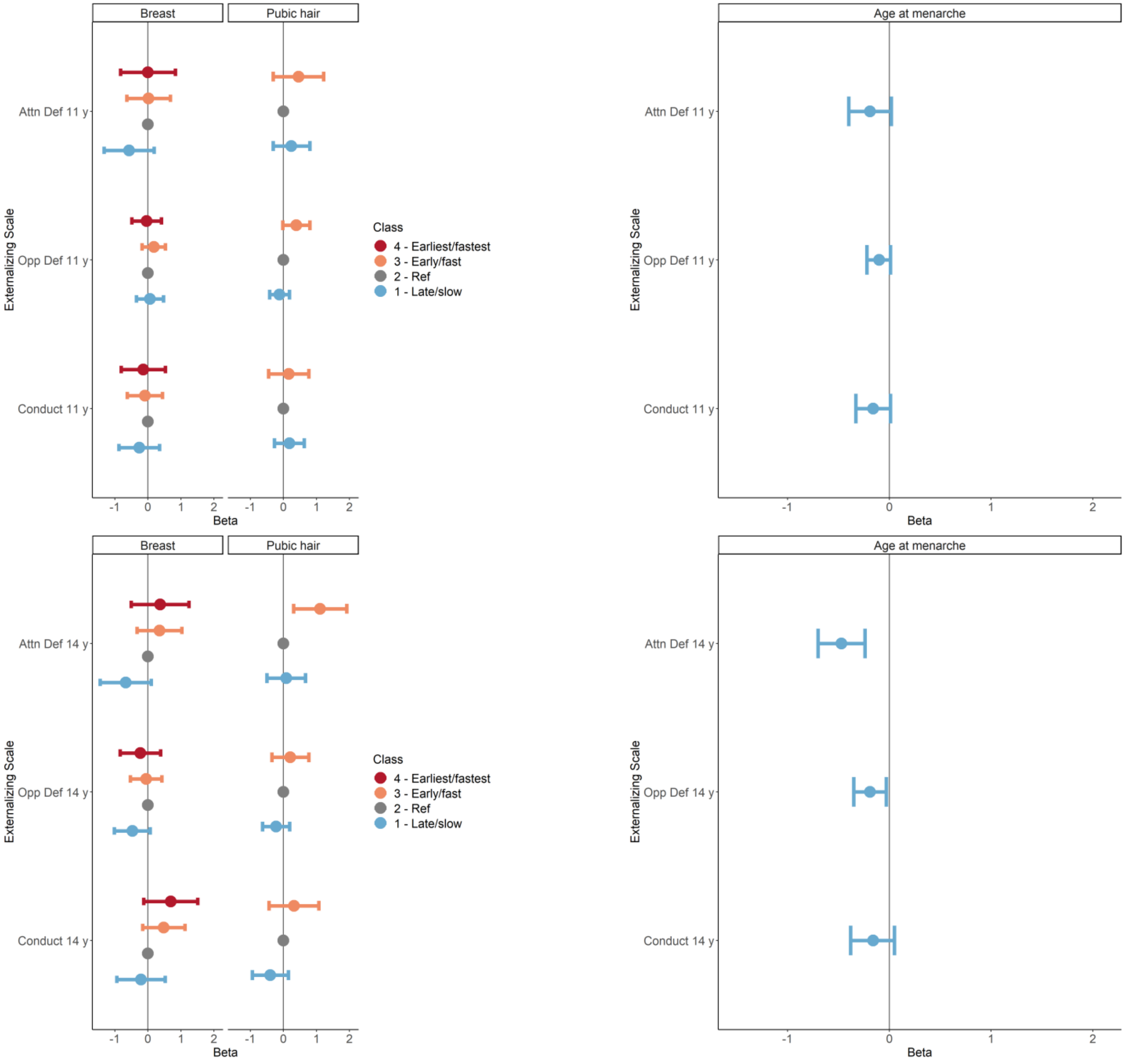

Supplemental Figure S8. Adjusted associations of female breast and pubic hair development classes and age at menarche with eating attitudes at ages 13 and 17 y

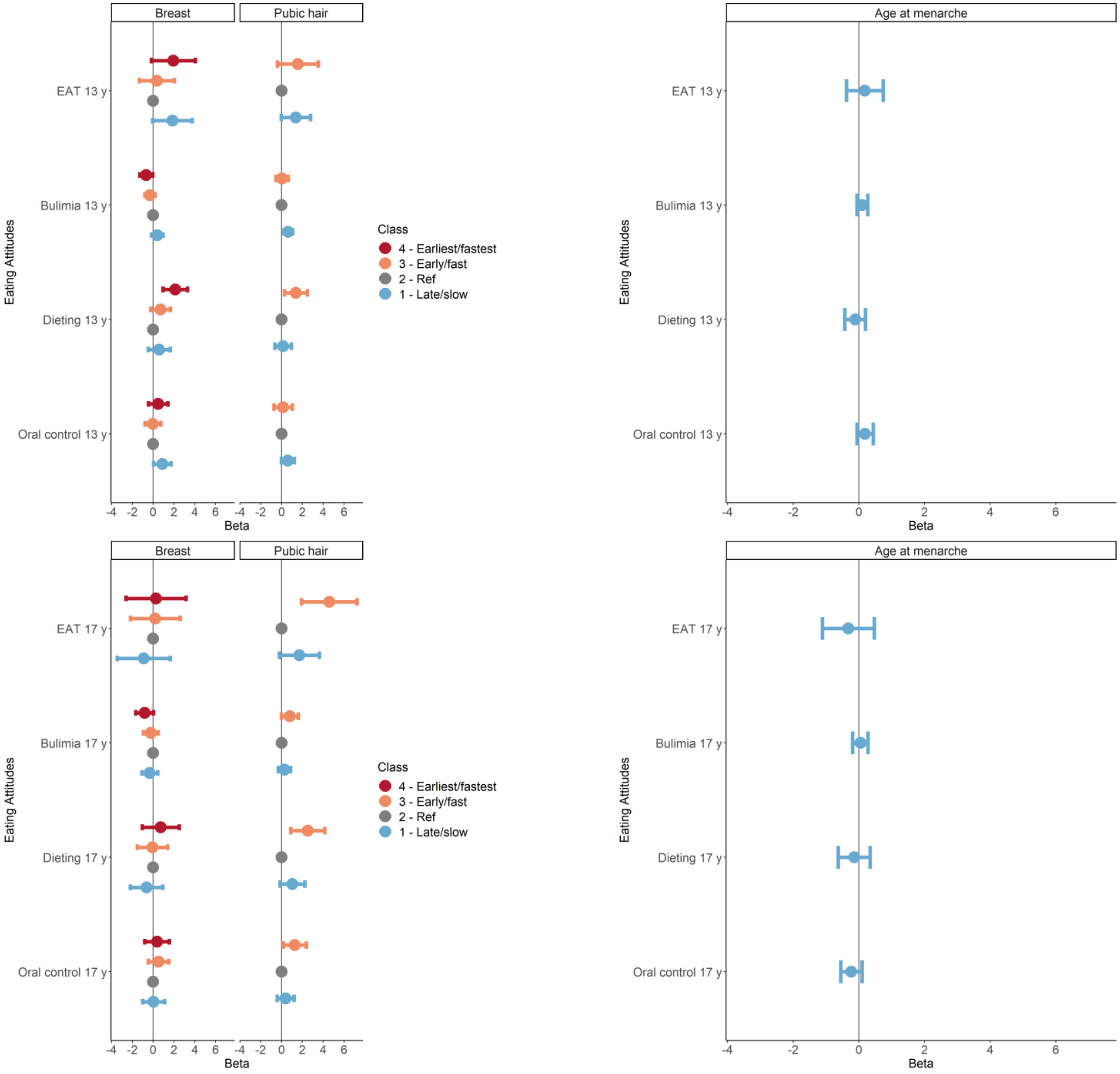

Supplement: Supplementary Tables and Figures [file mmc1.pdf]
